# Applying the ORBIT model to design a patient-facing mHealth app for diabetes prevention in urban primary care: translating patient perspectives into app design

**DOI:** 10.1093/tbm/ibag065

**Published:** 2026-09-22

**Authors:** Cara Stephenson-Hunter, Katherine Florian, Chandra Singh, Earle C Chambers, Andrew Telzak, Linda C Gallo

**Affiliations:** Department of Family and Social Medicine, Albert Einstein College of Medicine, Bronx, NY, United States; Department of Family and Social Medicine, Albert Einstein College of Medicine, Bronx, NY, United States; Department of Family and Social Medicine, Albert Einstein College of Medicine, Bronx, NY, United States; Department of Family and Social Medicine, Albert Einstein College of Medicine, Bronx, NY, United States; Department of Family and Social Medicine, Albert Einstein College of Medicine, Bronx, NY, United States; Department of Psychology, San Diego State University, San Diego, CA, United States

**Keywords:** prediabetes, mHealth, lifestyle behavior change, primary care, safety-net settings

## Abstract

**Background:**

Prediabetes affects nearly one in three U.S. adults-often progressing to type 2 diabetes within 10 years. Although prediabetes is increasingly identified in primary care and prevention strategies exist, uptake remains low, particularly in urban safety-net settings. This translational gap reflects the complexity of prevention, which is preference- and context-sensitive. Mobile health could bridge this gap and improve engagement, but effective design requires understanding patient needs, barriers, and care context.

**Purpose:**

Guided by the Obesity-Related Behavioral Intervention Trials (ORBIT) model, this formative study identified perspectives on prediabetes and diabetes prevention and translated them into components of a patient-facing digital intervention.

**Methods:**

Semi-structured qualitative interviews were conducted among 24 adult primary care patients with documented prediabetes in an urban safety-net health system. Interviews explored understanding of prediabetes, decision-making about prevention options, motivation for lifestyle change, and integration of behavioral changes into daily routines. Interviews were transcribed and analyzed using rapid qualitative analysis.

**Results:**

Participants (mean age 58.8 years; 83% women; 46% African American/Black; 46% Hispanic/Latino) described six interrelated themes: understanding the diagnosis; making informed treatment decisions; integrating lifestyle change into daily life; finding motivation; staying on track with behavior change; and navigating prevention within health and financial constraints. Themes revealed gaps in risk understanding, counseling, and action planning and were mapped to preliminary app features.

**Conclusions:**

This study translated patient-identified needs into initial components for a patient-informed mHealth tool. The findings define early-stage content domains and behavioral targets that will inform prototype refinement, usability testing, and evaluation in primary care.

## Teaser

Adults with prediabetes in an urban safety-net setting described gaps in knowledge and support after diagnosis, including uncertainty about their condition and limited awareness of prevention options. These findings informed the design of a mobile health tool to support initiation and sustained engagement in diabetes prevention in primary care.

Implications **Practice:** Patients with prediabetes may benefit from early support following diagnosis that improves understanding of prediabetes and prevention options and provides practical guidance for initiating and sustaining behavior change.

**Policy:** Health systems should address gaps between identifying prediabetes and supporting prevention by establishing systematic processes for risk communication, discussion of prevention options, and connection to evidence-based resources.

**Research:** Patient-informed formative research can identify intervention needs and guide mHealth development; consistent with the ORBIT model, interventions developed from these findings should undergo iterative refinement and preliminary testing before efficacy evaluation.

## Introduction

Type 2 diabetes mellitus (T2DM), affects approximately 30 million people in the United States, and its prevalence continues to rise [1]. Prediabetes—the stage at which blood glucose levels are abnormally high, while not elevated enough to be considered T2DM, increases the risk of progression to T2DM. An estimated 98 million people in the United States have prediabetes; without intervention, 25% will progress to T2DM within five years [2]. T2DM and prediabetes disproportionately affect racial and ethnic minority populations and people living in low-income urban communities. In these settings, social and structural determinants of health, including poverty, limited access to healthy foods, unsafe neighborhoods, and constrained healthcare resources, undermine prevention efforts and contribute to excess morbidity, mortality, and healthcare costs.

Evidence-based strategies such as the National Diabetes Prevention Program (DPP) lifestyle intervention and pharmacologic interventions, such as metformin, can substantially delay or prevent progression from prediabetes to T2DM, yet referral, uptake, and sustained engagement remain low, particularly in safety-net settings [3–5]. Safety-net health systems serve a disproportionate share of publicly-insured, un-/under-insured, and patients with limited resources. These settings are often characterized by high patient volume, competing clinical demands, complex multimorbidity, limited visit time, and constrained access to ancillary prevention resources [6–8]. In prior work, we identified several reasons why discussions about prediabetes and prevention are frequently missed or deferred in primary care within these settings, including time-constrained visits, variability in patient health literacy and readiness, and primary care providers (PCPs) limited awareness of available prevention options [9, 10]. These factors, combined with patients’ competing health and social needs [11] and financial and logistical barriers [12], contribute to delays in initiating evidence-based next steps following diagnosis of pre-diabetes.

Because the primary care setting is central to prediabetes diagnosis and management, and PCP recommendations strongly influence patient uptake, tools are critically needed to support timely action planning within routine care [13–15]. This need is particularly salient in safety-net settings and among groups historically excluded from prevention. This includes men, who tend to have less frequent primary care visits, are less likely to enroll in or complete lifestyle-based programs and may also be perceived by PCPs as less interested in preventive interventions [16, 17].

Health information technology (HIT) and mobile health (mHealth) tools offer an opportunity to extend prevention support beyond the clinic by reinforcing PCP guidance, supporting shared decision-making, and translating recommendations into actionable next steps [18, 19]. App-based approaches may be particularly well suited to engaging previously unengaged groups by providing flexible, self-directed, and potentially stigma-reducing pathways to prevention that reduce reliance on traditional group-based formats and frequent in-person visits [20–22]. However, most existing mHealth tools are not designed around the lived experiences of adults with prediabetes in low-income urban settings and are rarely integrated into primary care workflows [23, 24]. Grounding mHealth development in patient-centered discovery and behavioral science therefore offers an opportunity to create tools that meaningfully complement primary care and address persistent gaps in diabetes prevention in safety-net systems.

The present study builds on our prior qualitative work with PCPs which identified communication and workflow barriers to diabetes prevention [25]. Guided by the NIH Obesity-Related Behavioral Intervention Trials (ORBIT) model [26], we aimed to characterize patient needs, barriers, and facilitators influencing engagement in diabetes prevention and to translate these findings into design requirements for a scalable digital health tool to support communication, decision-making, and action planning to address pre-diabetes in primary care.

## Methods

All study procedures were approved by the Institutional Review Board (IRB) at Einstein-Montefiore. Participants provided electronic informed consent prior to participation. This study is reported in accordance with the Consolidated Criteria for Reporting Qualitative Research (COREQ) guidelines [27].

*Research team and reflexivity:* All interviews were conducted by C.S-H, a female health psychologist with formal training and more than 10 years of experience conducting qualitative interviews and research. C.S and K.F, a medical student and nursing student, respectively, were present during a subset of interviews and participated in coding and thematic analysis. The interviewer was involved in the development of the study and had an interest in understanding patient perspectives on diabetes prevention and informing the development of a patient-facing mHealth intervention. Participants were informed of the purpose of the study and the interviewer’s role in the research. No relationship between the interviewer and participants had been established prior to study participation. Reflexivity was supported through regular discussions among members of the research team during data collection and analysis, including discussion of emerging interpretations and potential sources of researcher influence and bias. The analytic team with all three members, met regularly to discuss emerging findings, compare interpretations, and resolve discrepancies in coding.

### Setting and participants

The study was conducted within an urban academic health system serving predominantly racial and ethnic minority and under-resourced communities in the Bronx, New York. Eligibility for the study was determined in two ways. First, we queried our primary care clinics’ electronic health record (EHR) systems to identify patients aged 18–74 years with evidence of prediabetes, no diagnosis of diabetes, and at least one primary care visit within the past eighteen months. We searched for ICD-10 codes consistent with prediabetes or abnormal glucose metabolism, including R73.01 (impaired fasting glucose), R73.02 (impaired glucose tolerance), and R73.9 (elevated glucose, unspecified). Because prediabetes is not always consistently coded in billing data, we also reviewed the EHR problem list to capture patients with documented prediabetes that may not have been reflected in diagnosis codes. Participants were recruited using convenience sampling from two sources. First, EHR systems were queried to identify potentially eligible primary care patients, who were invited to participate through study outreach. Second, potentially eligible patients were referred to the study team by health educators within the health system. All interested patients were contacted by telephone to confirm eligibility prior to enrollment. During this screening, participants were asked whether they had ever been told by a healthcare provider that they had prediabetes or were at risk for diabetes. Only individuals who reported prior awareness of their prediabetes were included, consistent with the study focus and to avoid nonclinical study staff being the first to disclose this diagnosis.

### Study design

The NIH ORBIT model [26, 28] provides a phased framework for the development and testing of behavioral interventions, progressing from intervention design (Phase I), to preliminary testing (Phase II), efficacy testing (Phase III), and effectiveness testing (Phase IV). Phase I includes defining the intervention’s behavioral and clinical targets (Phase Ia) and refining intervention components (Phase Ib) before preliminary testing. Consistent with Phase Ia (Define), the present study used formative qualitative research to identify patient needs, motivations, and barriers relevant to diabetes prevention and inform intervention development.

Complementary behavioral theories were selected based on their relevance to the patient-level determinants targeted by the intervention and their potential to inform actionable intervention strategies. The Theory of Planned Behavior [29] informed consideration of attitudes, perceived social norms, and perceived behavioral control related to diabetes prevention. Self-Persuasion Theory [30] informed strategies to elicit patients’ own motivations and reasons for behavior change, while Behavioral Economics [31] informed approaches to reducing barriers to action and supporting decision-making. A guided locus-of-control reframe [32] informed strategies to support patient agency while acknowledging health, social, and financial constraints that may affect prevention behaviors. These frameworks were used to interpret the qualitative findings and inform intervention design rather than as a priori categories for qualitative coding.

### Data collection

Participants were *N* = 24 adult, English-speaking primary care patients with EHR-documented prediabetes. The interview guide explored participants’ experiences after learning that they had prediabetes including their understanding of their diagnosis, decision-making regarding prevention options (such as the DPP or metformin), motivation and readiness for lifestyle change, integration of behavior changes into daily routines, and challenges related to health, social or financial circumstances. Findings from our prior study that interviewed providers within the same health system informed both the patient interview guide used in the present study and the interpretation of study results [9]. A brief demographic survey captured participant age, sex, race and ethnicity, education level, employment status, and prediabetes knowledge.

Participants were offered the option to complete interviews either in-person or virtually, according to their preference; all participants elected to complete interviews virtually. Virtual interviews were conducted using Zoom (Zoom Video Communications, Inc., San Jose, CA, USA) or Google Voice and lasted approximately 30–45 minutes. Member-checking was performed, and participants were not invited back for repeat interviews. All interviews were audio recorded, professionally transcribed, and de-identified for analysis. Transcripts were not returned to participants for review or correction, and participants were not invited back for repeat interviews. Interviews continued until thematic saturation was achieved, or no new themes emerged.

### Data analysis

A rapid qualitative analysis (RQA) [33] approach was used to synthesize findings systematically. RQA was selected because the study was designed to identify actionable patient needs and barriers to inform intervention development within ORBIT Phase Ia, and its structured approach facilitated the translation of qualitative findings into intervention design requirements. Analysis was primarily deductive, with domains informed by the study aims and semi-structured interview guide. A case-by-domain matrix was created to summarize participant responses across key domains. Three raters independently coded the first three transcripts to establish interrater agreement, after which remaining transcripts were independently coded and reviewed collaboratively to ensure consistency. Themes were identified, summarized, and refined through iterative team discussions. Researchers completed field notes and member checking was conducted, at the end of a subset of interviews to assess whether the findings reflected participants’ experiences and perspectives. Findings were interpreted using Designing for Dissemination and Sustainability (D4DS) [34] principles to translate thematic insights into app design requirements emphasizing scalability, literacy appropriateness, and alignment with patient preferences. Following qualitative analysis, findings were reviewed by the study team to identify patient needs and corresponding opportunities for intervention. Themes were not mapped one-to-one to individual modules; rather, findings across themes were considered collectively to identify cross-cutting design requirements. These included the need for accessible information about prediabetes and prevention options, support for personally relevant decision-making and motivation, practical strategies for initiating and sustaining behavior change, and content responsive to health and financial constraints. These requirements, together with the behavioral frameworks and D4DS principles.

## Results

### Participant characteristics

A total of 24 participants completed interviews, at which point thematic saturation was reached. Demographic characteristics of the participants are detailed in Table 1. The mean (SD) age was 58.8 (SD = 11.6) years, and 83.3% were women. Nearly half identified as African American/Black (45.8%) and 45.8% as Hispanic/Latino. More than half (54.2%) had completed postsecondary education, while 37.5% had a high school education or less.

**Table 1:** Participant demographics (*N* = 24).

Characteristic	*n* (%)
Gender	
Women	20 (83.3%)
Men	4 (16.7%)
**Race and Ethnicity**	
African American/Black	11 (45.8%)
Hispanic/Latino	11 (45.8%)
White	2 (8.3%)
**Country of Birth**	
United States	19 (79.2%)
Other	5 (20.8%)
**Education level**	
Less than high school	5 (20.8%)
High school or equivalent	4 (16.7%)
Associate degree/technical certification	4 (16.7%)
College degree (BA/BS)	4 (16.7%)
Graduate degree	5 (20.8%)
Missing/Not reported	2 (8.3%)
**Employment status**	
Employed full or part-time	13 (54.2%)
Retired	5 (20.8%)
Not working^a^	4 (16.7)
Other	2 (8.3%)

Participants who identified as Hispanic/Latino did not select a race category, although participants were allowed to select more than one race or ethnicity category. United States includes Puerto Rico and other U.S. territories.

a Includes participants who were unemployed, students, or receiving disability benefits.

### Thematic findings

Interviews revealed six interrelated themes that illustrate the complex realities of living with prediabetes and navigating existing prevention efforts: Understanding the Diagnosis; Making Informed Treatment Decisions; Integrating Lifestyle Changes into Daily Life; Finding Motivation; Staying on Track with Lifestyle Change; and Navigating Prediabetes in the Context of Health and Financial Realities. Themes and subthemes are presented below; representative quotes and corresponding implications for intervention design are provided in Table 2. Quotations are identified by participant gender (M = man; W = woman) and study ID; additional participant characteristics, including age and race/ethnicity, are provided in Table S1.

**Table 2:** Themes, subthemes, representative quotes, and design implications.

Theme	Subthemes	Representative quotes	Design requirements informed by findings
**1. Understanding the diagnosis**	Illness beliefs and representation; uncertainty about causes of prediabetes	“I don’t know like the science behind diabetes, like, I know, it’s like glucose and that stuff. But like I didn’t know.” (1002) “I mean, it’s 1 step away from being diabetic. That’s how I think when I hear prediabetes, it’s like, okay, next…we’re going to put you on medication.” (1012)	Include plain-language educational content explaining what prediabetes is, what causes it, and how progression to diabetes can be prevented or delayed. Use simple visuals and myth-busting interactive content to counter fatalistic beliefs (e.g. that medication or diabetes are inevitable).
**2. Making informed treatment decisions**	Treatment knowledge; decision support; provider communication	“…it’s like they don’t go into depth about what options you have… It is like you’re stuck trying to do it all yourself.” (1001) “No referrals to a health educator… just told to cut down on sweets.” (1005)	Provide accessible explanations of prevention options including lifestyle programs and medications, with tools that support shared decision-making to help users prepare for and advocate in provider conversations
**3. Integrating lifestyle changes into daily life**	Understanding glucose and diet; meal preparation; self-regulation challenges	“It’s a lot of hard work… You get the news and you gotta change your whole life.” (1011) “I’m stuffing myself with certain fruits that I just found out have a high level of sugar. So that makes no sense. Just like what am I supposed to eat?” (1005) “They tell you that [it]is the sweets, and…it’s starch, and the MSG. And all of that. But it’s hard to avoid” (1010) “ Yeah, I lost weight…now I’ve been gaining it again because I started working, and I’ve been snacking so I might be messing up because I keep buying a lot of junk to get me through the day, because its so boring. If I’m busy I won’t eat.” (1004)	Include practical dietary tools such as a food label scanner, ingredient-based recipe suggestions, and nutrition tracking. Offer culturally relevant meal ideas. Provide non-stigmatizing guidance on emotional eating and cravings, with signposting to relevant professionals (e.g. dietitians, mental health providers)
**4. Finding motivation**	Avoiding illness and complications; preserving personal goals and family roles	“…I have family members… that have passed away through the diabetes. I don’t want that life because … you’re used to living a certain way, and sometimes people don’t know how to change, and then they get amputated, and they have no fingers. I don’t live my life like that” (1002) “I want to be the best me I can be at any age…I’m not done… there’s still more life to live. I like to travel so, so you have to be in good health” (1008)	Enable users to identify and store personal health motivations and goals, including family roles, avoiding complications, and life aspirations, and surface these through periodic reminders tied to their stated reasons for change.
**5. Staying on track with lifestyle change**	Tracking tools; social and peer support	“The tracker helped me… I logged a McDonald’s meal… it damn near took all my points for the whole day… I haven’t been back since” (1018) “We take walks like during lunch, and we wear our watches, and we track each other…” (1014) “I get weak, so I don’t have…strong willpower … the group of ladies I work with now is amazing, because they hold me accountable.” (1011)	Incorporate self-monitoring tools for diet and physical activity. Include optional social features that allow users to share progress or goals with chosen contacts, supporting the peer accountability that users identified as key to sustaining behavior change.
**6. Navigating prediabetes within health and financial contexts**	Concurrent health conditions; financial barriers; food environment	“I think my real struggle is because I’m going through menopause. So it’s harder for me to drop the pounds.” (1003) “Living paycheck to paycheck at the moment … trying to keep it all together, working long hours, worrying about bills; [blood]pressure’s been high…mostly from stress…So, I need to deal with that first. (1019)” “It would be so great not to have to…buy expensive sort of old fruits and vegetables, you know, … [to have] one of those nice farmers markets. (1016)	Help users recognize when competing health priorities largely share the same management strategies (e.g. stress reduction, healthy eating, physical activity) and present these as a unified, actionable plan with concrete steps suited to their real-world constraints. Support navigation of relevant providers (PCPs, OB/GYNs, endocrinologists, social workers, community health workers) with tools to raise interconnected concerns in appointments, advocate for referrals. Include location-based tools to identify affordable food sources, safe activity spaces, and community resources, framed around what is feasible within the user’s environment and circumstances.

### Theme 1. Understanding the diagnosis

Many participants described confusion or uncertainty about what prediabetes meant for their health and how it related to their everyday behaviors. Although some understood prediabetes as a reversible condition requiring active management, they were often unclear about why their glycosylated hemoglobin (A1C%) was elevated, particularly when they believed they were already eating “healthy.”

**1a. Illness beliefs and representation.** For some, prediabetes felt abstract because it was asymptomatic, which weakened perceived urgency and made the diagnosis challenging to accept. As one participant stated, “They want to be convincing me that I’m pre-diabetic… but I don’t feel anything” (W, 1009). Others interpreted long-term prediabetes without progression as evidence that it was not an immediate threat, noting, “[been prediabetic] About 23 years… if I wasn’t doing good, I would have diabetes by now.” (M, 1019). In contrast, some participants framed prediabetes as a warning sign and described concern about whether it would inevitably progress, as reflected in “Okay, wait a minute, this could, this could be pre, but it could be pro, you know, somewhere down the line.” (W, 1008). Other participants also framed diabetes as uniquely threatening based on lived experience and feared complications, emphasizing, “I know diabetes is a very dangerous thing…if I have a choice in diabetes and cancer, I would rather the cancer… diabetes is very hard.” (M, 1019). Together, these illness representations shaped whether participants viewed prevention as necessary, urgent, and personally relevant.

**1b. “How did this happen?”** Participants often tried to make sense of what caused their diagnosis. Several described confusion when their beliefs about their sugar intake did not match the diagnosis. For example, participants stated, “I’m not the most in-shape guy, but I don’t eat crazy. I’m not a candy or soda person…So I was just confused, like how did this happen?” (M, 1018), and “I thought it was because…you ate like a lot of sugary foods. I really don’t eat sugary food.” (W, 1002). Another person attributed risk to a specific exposure and framed it as temporary, “I blamed it on a trip I made to the Dominican Republic…and over there I was eating a lot of starch… and a lot of sweets” (W, 1005). Other perceived gaps in information limited patients’ ability to interpret their risk. One expressed frustration with not receiving specific information she felt would help her understand her condition: “like how high is the prediabetes right now? I don’t know, because…they didn’t tell me that.” (M, 1010). These narratives suggest that limited understanding of etiology and A1C results may leave patients uncertain about which behavioral changes are necessary to reduce their risk of progression to diabetes.

### Theme 2. Making informed treatment decisions

Participants described uneven access to information about prevention options and variable experiences with clinical communication. These differences influenced whether they felt equipped and empowered to decide how to prevent diabetes.

**2a. Treatment knowledge.** Many perceived limited resources for people with prediabetes and reported low awareness of prevention programs such as the DPP. One participant expressed frustration, stating, “there’s…not much help for… people that’s pre-diabetic. You don’t hear about the programs… you gotta wait till you get sick to hear about them.” (W, 1001). Most reported never being offered lifestyle change interventions or programs.

Pharmaceutical options were also infrequently discussed. When medications such as metformin or weight-loss drugs were mentioned, participants often described uncertainty or concern shaped largely by personal or family experiences. For example, one participant expressed fear of side effects from Wegovy, explaining, “I’m afraid of the side effects…” and describing how a family member experienced “a lot of side effects … she lost [her] hair almost all.” (W, 1014). Others referenced perceived long-term harms. One participant stated, “My dad, he drinks that kind of medicine [metformin], and that’s no good in the future for you.” (W, 1007). More broadly, some expressed skepticism toward medication use in general, reflecting mistrust and a preference for non-pharmacologic approaches: “I’m not so much into medications like a lot of doctors just want to shove medication [at you].” (M, 1010). Together, these concerns, combined with limited exposure to structured prevention programs, contributed to a perception that few acceptable or trustworthy treatment options were available and reinforced a preference attempting independent lifestyle changes before considering medication.

**2b. Decision support and autonomy.** Participants described wanting more immediate, actionable guidance following diagnosis along with greater support for decision-making. One participant described delayed access to nutrition support following her prediabetes diagnosis, “it took me a year and a half to get a nutritionist. So in that year and a half, what could I have done by myself” (W, 1002), and another emphasized the need for real-time education “better than just giving a pamphlet…[and] good luck with that.” (W, 1004). Some participants described the absence of, or long delays in, follow-up planning following labs confirming prediabetes, “Nobody called me and said, ‘Hey, you’re prediabetic, this is what we’re going to do.’” (W, 1002). These experiences suggest that lack of decision support can translate into stalled action even when motivation is present.

**2c. Provider relationships and communication.** Trust and communication quality with health care providers seemed to significantly shape engagement. Positive relationships were characterized by collaboration and respect, “We have a partnership…her approach was, what can we do to get this [weight/AIC] down?” (W, 1003), and perceived caring where “[I] can feel like… somebody actually cares about if I’m gonna get through this” (W, 1011). Participants valued clear explanations and detailed recommendations, including documentation that reinforced understanding, “my doctor…she’ll let me know…this is what this means…Do you have any questions?…they put the notes in my chart…very detailed” (W, 1008). By contrast, others voiced a desire to be less actively engaged but expected clinician prompting, “I am not gonna volunteer no question until they [provider] ask…That’s what it’s supposed to be.” (M, 1019). These findings highlight how communication style and needs vary and how continuity can either support or limit informed engagement in prevention.

### Theme 3. Integrating lifestyle changes into daily life

“It’s a lot of hard work… You get the news and you gotta change your whole life.” (W, 1011).

Participants described lifestyle change as a process of translating abstract or generic guidance into concrete decisions within the constraints of everyday life. Rather than resistance to change itself, participants highlighted challenges related to understanding how food affects glucose, applying recommendations within work and cultural routines, and managing cravings, stress, and emotional triggers. These experiences underscore that sustaining diabetes prevention behaviors requires practical, personalized support that helps individuals make sense of guidance and adapt it to their lived contexts.

**3a. Understanding glucose, dietary triggers, and the glycemic index.** Many participants described uncertainty about what “cutting back” meant in practice and struggled to identify hidden sugars and carbohydrate sources. “I’m not eating candy…I’m not drinking soda but I’m eating other stuff that I’m thinking are healthy and they’re really not.” (W, 1005). Participants wanted more specific education, “Teach me how to do the macros. Explain the difference between carbs and sugar.” (W, 1001), and described confusion when dietary changes produced unexpected results, “every time I change [my] diet the A1c might drop, but then the triglycerides go up…If I’m eating right, why is all this happening?” (W, 1001). Others described learning that sugar extends beyond sweets, “I thought it was just candy… but then [the doctor] said certain foods have sugar too…” (W, 1004), and uncertainty about what to eat, “maybe I should juice. But I’m like, isn’t that sugary?” (W, 1002). These accounts reflect a need for practical, literacy-appropriate education that translates nutritional guidance into everyday choices.

**3b. Meal preparation and substitutions.** Participants described difficulty applying dietary guidance in daily routines shaped by work schedules and cultural food practices. One participant explained a demanding work routine, “I work every day…construction… I might eat…half [a sandwich] and some water. That’s it for the day until I go home” (M, 1010). Others emphasized cultural food preferences and identity, “I eat a lot of like yams and boiled bananas. I’m Puerto Rican… I grew up eating that stuff.” (W, 1004). Participants wanted realistic meal planning guidance rather than vague rules, as reflected in “Add like a meal plan…an example of what eating healthy is” (W, 1002) and “Don’t show… all this food, but help prepare or plan a meal…according to your particular needs” (W, 1001). These findings support the need for culturally relevant substitutions and practical meal planning supports.

**3c. Self-regulation challenges.** Participants described cravings, emotional eating, and unstructured snacking as barriers to self-regulation. One participant noted struggling with stress-related binge eating following a traumatic event, “If I stay home, I might binge eat because I’m thinking about the accident.” (W, 1003), while another acknowledged succumbing to cravings of high-sugar foods despite awareness, “I love that milkshake… it got a lot of sugar, but I drink it anyway” (W, 1009). Several described struggles with cravings and willpower, “I feel confident. But… I know me… it’s hard sometimes not to feed into those cravings” (W, 1011) and “I suffer from sweet tooth… I need sugar all the time, and the answer was just ‘stay away from sugar.’” (W, 1006). These experiences indicate that behavior change support must address cravings, emotional triggers, and setbacks using nonjudgmental strategies rather than simple avoidance messages.

### Theme 4. Finding motivation

Participants described both prevention-focused motivation (avoiding illness) and promotion-focused motivation (living well and meeting life goals), often grounded in family experiences.

**4a. Avoiding illness and fear of progression.** Fear of diabetes complications was a prominent motivator. Many cited family experiences and feared outcomes such as dialysis, “My aunt is on dialysis. I don’t want to end up like that.” (W, 1005), and recalled learning about complications early in life, “my mother…would tell me about people who had gangrene from diabetes.” (W, 1016). Others described motivation through a desire to avoid medication and organ damage, “I’m trying not to end up having to take medication.” (W, 1011) and “I also have a lot of motivation. If you don’t [act], it’s gonna damage your kidneys…damage your whole system.” (M, 1019). However, some participants monitored A1C as a threshold and minimized concern until it crossed into diabetes, “as long as I didn’t cross over to the diabetic range… I didn’t worry about it.” (W, 1021). This pattern suggests that motivation may depend on how risk is framed and whether progression feels imminent.

**4b. Living a desired life.** Many participants described motivation linked to identity, function, and future goals. One participant emphasized her desire to remain active and travel, “I want to be the best me… I like to travel…you have to be in good health in order to have that lifestyle.” (W, 1008). Others expressed motivation rooted in caregiving. and modeling, “I need to show [my children] that…they need to make better choices” (W, 1004), and fear of burdening family, “I don’t want to put my children and my grandchildren through anything like that…So I figured that I have to…make a change.” (W, 1003). Participants also described motivation linked to weight and reproductive goals, “feels good…going to stores…buying like a [size] 16…(W, 1006) I’m …trying to have another baby” (W, 1004). These narratives highlight the importance of helping patients connect prevention behaviors to personally meaningful goals.

### Theme 5. Staying on track with lifestyle change

Participants described tools and social support that helped them maintain behavior change through feedback, accountability, and shared experience.

**5a. Tracking and monitoring tools.** Participants valued tools that supported self-monitoring and helped them learn how foods affected their body or how to monitor their physical activity. Some described interest in activity tracking, “counting your steps is a thing so incorporating that” (W, 1002). Others emphasized glucose monitoring as transformative, “what changed my life…was the monitoring your own sugar…there’s an app on my phone…[having this earlier] would have started me off in the right track” (W, 1012). Participants also described using scanning apps in grocery stores, “I’m scanning everything…It tells you, if it’s good or bad…gives you a more healthier option” (W, 1013), and noted how tracking promotes accountability, “trackers are amazing, because you have to hold yourself accountable” (W, 1011). Seeing personalized data was described as more persuasive than education alone. One participant reflected that although they had been advised to reduce sugar, using a continuous glucose monitor helped them recognize that foods not typically considered “sugary” could have a greater impact on their blood glucose: “I did put 6 packs of sugar… and my levels didn’t change. But when I had the sandwich, it did change… so hearing it and seeing the numbers, it was something different.” (W, 1013). These findings support integrating simple feedback tools that translate individual behaviors into visible, personalized outcomes.

**5b. Social and peer support.** Participants described encouragement and accountability from peers, coworkers, friends, and family. For example, “my coworkers…on the same weight loss journey…We take walks…track each other” (W, 1014), and “the group of ladies I work with now is amazing, because they hold me accountable” (W, 1011). Others emphasized supportive friendships, “They’re my people. I want you to be around” (W, 1008), and family encouragement, “my youngest daughter…[said] mom, you got to take care of yourself. I’ll go to the gym with you.” (W, 1005). Participants also described peer experience as more impactful than clinician advice alone, “it’s better to hear it from somebody else that went through it…It affects you more” (W, 1013). These narratives suggest that social support features and peer normalization can strengthen sustained engagement.

### Theme 6. Navigating prediabetes in the context of health and financial realities

Participants described managing prediabetes alongside other health conditions and financial barriers that constrained their choices and access to care.

**6a. Concurrent health conditions.** Many participants described physical and mental health issues that complicated prevention efforts and lowered bandwidth for lifestyle change. One participant described the interplay of trauma and mental health, “I was in a domestic violence relationship…trying to make sure my daughters were good… I suffer from depression and anxiety…so that may have played into me not caring about a lot of things.” (W, 1004). Others described multiple diagnoses accumulating, “[you’re] going to turn 46…suffering from high blood pressure…They gave you medication and now this.” (W, 1005), and endocrine and life-stage barriers, “ever since…a thyroid condition, I’ve never been able to…lose weight” (W, 1002) and “because I’m going through menopause its harder…to drop the pounds.” (W, 1003). These experiences emphasize the need for interventions that acknowledge comorbidity and integrate realistic options given patient capacity.

**6b. Financial circumstances and food environment.** Participants described structural constraints related to the food environment, insurance coverage, and appointment access. One participant stated, “You can’t avoid all the chemicals in food in this country.” (M, 1010), and others highlighted cost barriers, “if healthy food is so great, why is all of this stuff so expensive.” (W, 1001). Participants described the desire for better food access, including farmers’ markets, “It would be so great not to have to…buy expensive…old fruits and veg…one of those nice farmers markets.” (W, 1016). Insurance barriers were also common, “[my doctor] was trying to get me… [weight loss medication] …but my insurance was denying it.” (W, 1003), and access challenges were described as systemic, “getting appointments is super difficult…Sometimes the insurance doesn’t cover, or the doctors are not participating” (W, 1012). These narratives underscore that prevention support must account for affordability and access constraints rather than assuming unlimited choice.

**Translation of findings to intervention design.** Across the six themes, findings identified several cross-cutting needs that informed development of the mHealth intervention. Uncertainty about prediabetes and limited awareness of prevention options informed the need for accessible education and decision support. Challenges translating lifestyle recommendations into daily life informed practical, personalized content addressing nutrition, cravings, and realistic behavior change. Participants’ descriptions of motivation, tracking, and social support informed strategies to connect prevention to personally meaningful goals and support continued engagement. Finally, experiences managing prediabetes within competing health and financial circumstances emphasized the need for intervention content that acknowledges patient capacity, affordability, and access constraints. These findings were considered collectively, rather than mapped one-to-one to individual modules, in developing the intervention content and components shown in Figure S1.

## Discussion

Using an ORBIT Phase 1a discovery approach, this study identified patient-derived design requirements for a patient-facing mHealth intervention to support diabetes prevention in urban safety-net primary care. Rather than serving only as a needs assessment, the study translated patient experiences into preliminary intervention components and functional priorities for app development. Findings show that effective digital prevention support must address how patients understand prediabetes, interpret HbA1c levels and risk, make decisions about prevention options, and sustain behavior change amid competing clinical, social, and financial demands.

These findings advance the field by showing that diabetes prevention barriers in safety-net settings are not limited to knowledge deficits or access constraints. They also reflect a mismatch between how prediabetes is communicated in primary care and how patients must act on that information in daily life. Participants requested plain-language explanations, culturally relevant guidance, practical decision support, and continuity of support beyond brief clinical encounters. This points to a clear translational opportunity for mHealth tools to extend counseling between visits, reinforce understanding, and support feasible action planning in real-world primary care settings.

We found that limited awareness of prediabetes, inconsistent counseling, and structural barriers constrain engagement with evidence-based prevention strategies. These findings are consistent with prior qualitative work conducted in two primary care practices, which showed that ethnically and racially diverse adult persons with pre-diabetes often lack knowledge of management options and overestimate their risk [35]. Researchers found that participants perceived brief, specific counseling as motivating initial action [35], yet our findings suggest that counseling alone is insufficient to support sustained prevention without ongoing, context-sensitive support. These prior studies also suggest that low uptake of prevention reflects not only knowledge gaps or access barriers but also a misalignment between how prediabetes is communicated in primary care and how risk is managed in everyday life. Participants did not simply need more information, they needed a coherent way to interpret HbA1C, understand causality, and connect lab results to feasible actions. Emerging evidence suggests that self-monitoring tools, such as continuous glucose monitors, can make these connections visible by allowing individuals with prediabetes to observe how behaviors like exercise influence glucose in real time, improving self-monitoring, goal setting, self-efficacy, and program adherence [36, 37]. This suggests that traditional brief counseling and referral-based approaches, while necessary, are often insufficient for supporting sustained prevention in safety-net contexts where patients face competing social and health demands.

Research on shared decision-making in chronic disease prevention emphasizes the importance of patient autonomy [38]. Prior studies indicate that patients with prediabetes often prefer lifestyle change as an initial approach and may view metformin as acceptable when it is recommended and explained by their PCP [35]. Notably, in our study few participants reported discussing medications such as metformin with their PCPs, despite low cost and ADA endorsement, and many relied on anecdotal, often negative accounts that reinforced medication avoidance. Our findings demonstrate that shared decision-making and patient autonomy requires more than the presence of options; it requires timely, tailored, and culturally grounded decision support. Participants wanted clear explanations of prevention options, realistic guidance that fit their routines and food practices, and continuity of support after diagnosis. When providers were able to engage collaboratively, patients felt more confident and supported in prevention; however, structural constraints in primary care limited the extent and frequency with which this could occur. National survey data suggest that these constraints are widespread, as PCPs report gaps in prediabetes management knowledge, low referral to lifestyle programs, and system-level barriers such as limited resources and insurance coverage, all of which restrict their ability to provide the sustained, collaborative support patients describe as essential [10]. This tension highlights a clear opportunity for a patient-facing mHealth tool to reinforce clinician guidance, scaffold shared decision-making, and maintain continuity of support between visits while preserving the central role of the PCP.

A meta-synthesis of qualitative studies found that, beyond perceived importance, motivation for diabetes prevention is strongly influenced by supportive relationships, enabling environments, and coping strategies that help individuals initiate and sustain lifestyle change [39]. The present findings underscore how dynamic and context-dependent these support factors are in real-world settings. Motivation was often grounded in family experiences and life goals, but sustaining behavior change depended on ongoing feedback, accountability, and practical tools that could adapt to work schedules, stress, comorbidities, and financial constraints. Together, these findings reinforce that effective diabetes prevention cannot rely on one-time counseling or referrals alone, but instead requires continuous, context-sensitive support that helps individuals identify, activate, and sustain both internal motivations and external sources of support.

### Design implications

Results of the current study will inform the specification of concrete design requirements for a patient-facing digital health tool, prior to prototype development and usability testing. First, educational content must demystify prediabetes using plain language and practical examples that clarify how everyday behaviors influence risk. Second, tools should support small, self-selected, and contextually feasible goals rather than prescriptive behavior change targets. Third, sustained engagement strategies such as reminders, progress feedback, peer or family involvement, and optional community-based supports are critical for maintaining motivation over time. Mapping these findings to app design targets (Table S1) illustrates how qualitative findings can inform tone, literacy level, personalization, and behavioral prompts in digital interventions. The Figure S2 provides additional examples of module content and app features, illustrating how the intervention will be delivered in practice.

### Implications for practice and future research

The next step in this research program is to apply these findings to develop and refine the mHealth intervention that supports shared decision-making and action planning for diabetes prevention in primary care. The intervention is designed to complement clinician recommendations by providing patient-centered education and guidance beyond time-constrained visits. Guided by the ORBIT framework, the design requirements generated in this Phase Ia study will inform Phase Ib usability testing with patients and PCPs, followed by Phase IIa and IIb studies to evaluate feasibility, engagement, and implementation within routine clinical workflows. Future work will also examine multilingual adaptations and strategies to integrate the intervention with community and clinical resources to support sustained diabetes prevention.

### Limitations and strengths

Several limitations of the current study should be acknowledged. The qualitative design limits generalizability; however, this approach allowed for an in-depth exploration of patient perspectives on prediabetes and diabetes prevention -a phenomenon that remains insufficiently understood, particularly in safety-net primary care contexts. By prioritizing depth over breadth, the study illuminated how patients interpret their diagnosis, navigate barriers, and identify supports that align with their lived experiences. The study was conducted at a single site within one health system, which may also limit generalizability. Yet this setting, serving one of the nation’s most socioeconomically, ethnically, and racially diverse patient populations, provided a unique opportunity to examine diabetes prevention through an equity lens. Insights from this population are therefore highly relevant to similar urban-based health systems across the United States.

The sample was predominantly female, which may limit the extent to which the findings capture the experiences and perspectives of men with prediabetes. Although the interview guide and recruitment materials were translated into Spanish, we were unable to recruit participants who were Spanish-speaking only or who spoke other languages. Therefore, all interviews were conducted with English-speaking participants, excluding individuals with limited English proficiency whose perspectives may differ. Nonetheless, the sample reflected the racial, ethnic, and socioeconomic diversity within the Bronx, offering a strong foundation for future studies to expand recruitment strategies and develop multilingual, culturally tailored adaptations of the intervention. Additionally, there is potential for selection bias, as individuals who chose to participate may have had either particularly positive or negative experiences with prediabetes care. Because participants were recruited based on provider-diagnosed prediabetes documented in the EHR, the sample may also reflect patients who were more likely to be screened, tested, or engaged in care, potentially excluding individuals at elevated risk who lack a usual source of healthcare or have not been formally diagnosed. However, participants in this study reported both favorable and critical views of their care and the support available for diabetes prevention. This range suggests that findings reflect diverse and balanced experiences.

## Conclusions

This study identified key patient needs, barriers, and facilitators influencing engagement in diabetes prevention among adults with prediabetes in an urban safety-net setting. Findings informed concrete design targets for a patient-facing digital mHealth tool that emphasizes clarity, autonomy, contextual fit, and sustained engagement. Together, these results provide a practical blueprint for developing culturally responsive, literacy-appropriate, and scalable digital interventions that bridge clinical and community contexts and support equitable diabetes prevention in primary care.

## Supplementary Material

ibag065_Supplementary_Data

## Data Availability

De-identified data from this study will be made available (as allowable according to institutional IRB standards) by emailing the corresponding author

## Welfare of Animals

This article does not contain any studies with animals performed by any of the authors.
