# Supplementary material for: Applying the ORBIT model to design a patient-facing mHealth app for diabetes prevention in urban primary care: translating patient perspectives into app design
Source: Transl Behav Med. 2026 Sep 22;16(1):ibag065. doi: 10.1093/tbm/ibag065 (PMC13596773; doi:10.1093/tbm/ibag065)
Supplement: Supplementary file 1 [file ibag065_supplementary_data.zip › Supp_Table_S1_Participant ID.docx]

**Supplementary Table S1.** Participant Characteristics by Study ID

| Participant ID | Gender | Race/Ethnicity | Age |
| --- | --- | --- | --- |
| 1000 | woman | AA/Black | 66 |
| 1001 | woman | AA/Black | 48 |
| 1002 | woman | AA/Black | 31 |
| 1003 | woman | AA/Black | 57 |
| 1004 | woman | His/Latino | 46 |
| 1005 | woman | His/Latino | 46 |
| 1006 | woman | AA/Black | 57 |
| 1007 | woman | His/Latino | missing |
| 1008 | woman | His/Latino | 72 |
| 1009 | woman | His/Latino | 55 |
| 1010 | man | AA/Black | 68 |
| 1011 | woman | His/Latino | 47 |
| 1012 | woman | His/Latino | 59 |
| 1013 | woman | AA/Black | 42 |
| 1014 | woman | His/Latino | 59 |
| 1015 | woman | His/Latino | 56 |
| 1016 | woman | White | 71 |
| 1017 | man | White | 69 |
| 1018 | man | His/Latino | 54 |
| 1019 | man | AA/Black | 70 |
| 1020 | woman | AA/Black | 71 |
| 1021 | woman | His/Latino | 70 |
| 1022 | woman | AA/Black | 71 |
| 1023 | woman | AA/Black | 70 |
