# Supplementary material for: Applying the ORBIT model to design a patient-facing mHealth app for diabetes prevention in urban primary care: translating patient perspectives into app design
Source: Transl Behav Med. 2026 Sep 22;16(1):ibag065. doi: 10.1093/tbm/ibag065 (PMC13596773; doi:10.1093/tbm/ibag065)
Supplement: Supplementary file 1 [file ibag065_supplementary_data.zip › Sup_Fig_S1.docx]

**Supplementary Figure S1. Mapping behavioral theories, participant insights, and mHealth intervention modules and features.** Modules and features were developed based on participant insights identified through qualitative interviews. Behavioral theories informed the translation of these insights into intervention content designed to support informed and sustainable behavior change.
